# Supplementary material for: A label’s a label, no matter the dog: Evaluating the generalizability of the removal of breed labels from adoption cards
Source: PLoS One. 2020 Sep 10;15(9):e0238176. doi: 10.1371/journal.pone.0238176 (PMC7482916; doi:10.1371/journal.pone.0238176)
Supplement: Attachment — Submitted filename: Response to Reviewers.docx [file pone.0238176.s002.docx]

**Response to Reviewers**

**Response to Editor Comments**

Specific comments

1. Lines 135 and 142 Since the study has already been completed, please change "aim" to "aimed”

*We have changed both instances to “aimed.”*

2. The paper will have an international readership, so reference to US-specific concepts that are not widely known internationally need to be accompanied by enough information to make them understandable. This applies to "501(c)(3)" on line 147 and perhaps also to "Good Samaritan" on line 191 (not clear to me if this term is used in the general sense of somebody doing a good deed for an unrelated individual in need, or if it refers to something more specific).

*Thank you for noticing this, we agree that these concepts may not be clear to international readership. We	removed “501(c)(3)” from the text as the descriptor “not-for-profit” was already	included. We changed “Good Samaritan” to “concerned citizen.” In addition, we added a new sentence to the Limitations and Future Directions section, starting at line 369: “This study was conducted in a New York City limited intake shelter in the United States. The results may not be as generalizable to shelters outside of the United States due to different policies, cultures and animal welfare approaches [4].”*

3. The first paragraph of the discussion is currently only understandable to somebody who has already read the previous text and retains important concepts such as LOS, LI and Bideawee's unique multi-location structure. Please consider rewriting, perhaps by adding a first paragraph which sums up the main results in a stand-alone way.

*Thank you for the constructive feedback on the opening paragraph of our	discussion. Reviewer 2, in their first specific comment, recommended removing	the abbreviations LOS, LI and OA for clarity and readability. We have removed	the abbreviations accordingly and agree it makes the text more readable. We	have also revised the first paragraph of the Discussion section to make it a	clearer, more stand-alone summary for readers.*

**Response to Reviewer 1 Comments**

Comments to the author

I found this a very interesting paper that opened-up some new questions around how information attached to shelter dogs can shape how long they stay in the shelter, or their Length Of Stay. I thought the paper was clearly written and a succinct argument and clearly formed argument through out. I think the only aspect I would liked to have seen a little bit more discussion or, perhaps in the conclusion is discussion of any research, or to pose research questions, that seek to explain some of the cultures around dog breed/colour type that means that the labelling can exacerbate entrenched assumptions about dogs of a certain breed or colour.

*Thank you to Reviewer 1 for their kind words and feedback. We have added in commentary on coat color to the Discussion section (starting at line 329) and believe the manuscript has been greatly improved. The section now includes the following: “Dogs with a brindle coat color were significantly less likely to be adopted relative to black dogs in the Breed Labels Used period, but there was no significant difference in time to adoption in the Breed Labels Not Used period, while those with a tan coat color were significantly more likely to be adopted relative to black dogs in the Breed Labels Not Used period, but there was no significant difference in time to adoption in the Breed Labels Used period. One possible reason for these inconsistent results is that dogs were grouped based on their primary color only – for example, both all-black dogs and black-and-white spotted dogs were classified as a black coat color if the black-and-white spotted dogs had a primary color record of black. Future studies could parse these color differences further to investigate additional linkages between coat color and breed labelling, especially since there are myths around both variables (e.g., “is ‘black dog syndrome’ exacerbated by breed labels?”).”*

**Response to Reviewer 2 Comments**

Comments to the author

Overall, this paper is excellently presented, with clear goals and rationale, a well designed data collection methodology, clearly written, and conclusions that do not over-interpret the results or over-extend their applicability. It addresses an important topic that requires more research, and contributes to the current literature by providing a generalisable analysis (a semi replication attempt) of a previously-published hypothesis, with a large sample size.

*We thank Reviewer 2 for providing us with such insightful comments and feedback on this	manuscript. Their assessment of our work was clear and invaluable during this revision	process. We have made all the Reviewer’s suggested changes. These changes have	substantially improved the manuscript and we are extremely grateful for the helpful	feedback.*

My only issue with the paper is that the statistical analysis has some clumsiness to it. Specifically, it's great that you used survival analysis, which is the natural way to analyse the data set, but you could have analysed the whole data set (i.e. including the breed label covariate) within one survival analysis model, despite the violation of the proportional hazards assumption. Survival analysis is a very mature field of applied statistical analysis, and there are a number of alternative survival analyses that would have been suitable, such as the accelerated failure time models (where you'd have modelled the probability of being adopted/still being in the shelter by day x). I think this is important to note because you discuss the different analysis options in both the methods and the limitations section (which I like), but I think you short change survival analysis modelling a bit by not explaining that there are models available to handle the violation of the proportional hazards assumption . For example, I think the class of 'longitudinal time-to-event' models that model a longitudinal data pattern (e.g. the dog's behaviour over time at the shelter) and an event (e.g. adoption) jointly would be a perfect way of analysing shelter dog data in a unified approach (e.g. see the review here https://doi.org/10.1146/annurev-statistics-030718-105048).

Because of the large sample size used, the fact that this is not a within-individual study, and the consistent patterns in the descriptive data showing the effect of breed labels, I think the current analysis is fine for publication (and wouldn't recommend a re-analysis) but I would recommend a few lines in the Discussion/Methods highlighting that you could have used a more sophisticated survival analysis model for your data, which would have reduced the number of models from three to one.

*Thank you for providing such thoughtful, detailed commentary on the statistical analyses. We appreciate the insight and recommendations for how to proceed with the analysis. We agree that the ‘longitudinal time-to-event’ model could be a particularly useful approach. As the reviewer does not recommend reanalysis, we have referenced the model in the Discussion section (line 348) and the Limitations and Future Directions section (line 384): “In addition, research into models such as time-to-event models and accelerated failure time models that make length of stay analyses easy to conduct and understand would be beneficial and drive further uniformity among models in future studies.”*

Finally, as mentioned in my specific comment below, it might also be a good opportunity to highlight the need for causal modelling tools in estimating the relationships between shelter behaviour, adoption times, breed etc. I'd recommend the accessible article by Rohrer (2018: https://journals.sagepub.com/eprint/mpV35gbHH9zAw8Zn2wh7/full) for an introduction to how observational data can mislead us. Obviously, this is just a personal preference, but I think it would add to the current literature on this subject, because shelter research is often faced with messy data about which we want to make causal claims.

*We have cited the work by Rohrer in the paper and added in commentary on the benefits of causal modelling of observational data in the Limitations and Future Directions section, starting at line 379, to encourage future studies to apply these valuable models: “Causal modelling of observational data could be used to provide further structure to data analyses and help animal welfare researchers determine which variables directly impact the independent variable (e.g., length of stay) and which variables indirectly impact the independent variable [25]. This could help streamline the number of variables animal welfare researchers examine and make the set of variables more uniform across studies.”*

Specific comments

4. Consider whether you need to abbreviate terms like 'length of stay', 'open admission', 'limited intake' -- for me, they obscure the prose and are not needed as they don't appear often enough.

*We agree and have reflected your suggestion. All abbreviations have been	removed.*

5. Line 14: parenthesis around the MW-U test results.

*We have added in parentheses.*

6. Line 33: "as a whole"?

*Correct, thank you for catching the typo. We have edited accordingly.*

7. Line 158: what did the data review comprise of, and why did more than 1000 records not meet the conditions for inclusion?

*The 3,525 records number comes from the total number of records from January 1, 2016 to June 30, 2019 in the Bideawee Pet Point system. Once the duplicate records and records not in the two 16-month time periods of February 1, 2016 to June 30, 2017 and February 1, 2018 to June 30, 2019 are removed, there are 2,508 records remaining for a further, in-depth review. This review, outlined in Figure 1, removed records where (1) the dog died or was euthanized for a medical condition; (2) the dog was returned – we only used the last record (where the adoption was successful) for dogs that were returned and therefore had multiple records; (3) there was a special case (e.g., one dog ran away); and, (4) where breed labels were not applied correctly. We see how this 1,000-record decrease could be confusing, so we have changed the “3,525 records” to “2,508 records,” referring to the number of records in the pertinent period, to clarify.*

8. Line 247: I'm not sure what discussing these other analysis options adds to the paper. Binning the length of stay into a binary variable (line 251) is almost certainly a terrible idea, as it will result in significance loss of power. Similarly, that length of stay is not normally distributed is not really a problem with modern day computing resources (and it is only a problem if one analyses data using a Guassian likelihood if the conditional distribution of the data given the model parameters deviates (far) from a Gaussian distribution). Your mention of 'parametric' models on Line 252 is a bit narrow, implying that parametric is synonymous with 'normally distributed' -- there are many likelihood functions suitable for length of stay-type data, as evidenced by the numerous types of parametric survival analyses.

*We agree with the reviewer and see how listing the other analysis options could detract from the paper. We have removed the commentary.*

9. Line 249: "cannot be less than zero" -> "cannot be less than one".

*Correct, thank you for catching the typo. We have edited accordingly.*

10. Line 281: Was the violation of the proportional hazards assumption the reason why you conducted the Mann-Whitney test? If so, I think the paper would benefit from stating this more clearly.

*We ran the Mann-Whitney test as a “step one” to see if there was a statistically	significant difference in the average and median lengths of stay with and without	breed labels. If there had not been a statistically significant difference, we would	have not progressed to the Cox regression model. To clarify this we have added in	the following for clarity at the beginning of the Statistical Analyses section: “The	first step in the analyses was to see if there was a statistically difference average	and median lengths of stay between the Breed Labels Used and Breed Labels Not	Used group. If there was significance, the impact of other variables on length of	stay would be evaluated to isolate the specific impact of breed label use.”*

11. Table 3: Can the 95% confidence intervals be presented as an inclusive range, e.g. 0.8 [0.7, 0.9] rather than 0.8 (0.7 - 0.9)? The latter implies the interval is exclusive, and could be mistaken for saying the numbers are being subtracted.

*We agree with the reviewer’s suggested comment and have changed the intervals in Table 3 to be presented as an inclusive range.*

12. Line 321: I think you should be careful in assuming that breed label use was driving the change based off the fact that behaviour was the only significant covariate in the model. Observational data analysis is extremely messy, and there are numerous unintuitive examples where observational data mislead us (see Pearl (2000) 'Causality' for more). It might be a good opportunity to highlight the benefits of such 'causal modelling of observational data' methods for shelter behaviour research, if it's something you are interested in.

*This is an extremely important point given the limitations regarding the correlational design. We have removed the following sentence from the Discussion section (line 321 of the original manuscript): “The fact that there was only one covariate in the model with a significant impact on LOS strengthens the prediction that the change in breed label use might have driven the change in LOS, rather than the reduced LOS being the result of other factors like change in population’s age groups or adoption site.” We then added in commentary on the benefits of causal modelling of observational data in the Limitations and Future Directions section, starting at line 379, to encourage future studies to apply these valuable models: “Causal modelling of observational data could be used to provide further structure to data analyses and help animal welfare researchers determine which variables directly impact the independent variable (e.g., length of stay) and which variables indirectly impact the independent variable [25]. This could help streamline the number of variables animal welfare researchers examine and make the set of variables more uniform across studies.”*

13. Line 356: This could be a good opportunity to highlight other methods of survival analysis that you could have used, e.g. accelerated failure time models, time-to-event models that do not consider a proportional hazards assumption.

*We agree with the suggestion and have noted these models in the Limitations*

*and Future Directions section (line 384): “In addition, research into models such	as time-to-event models and accelerated failure time models that make length of	stay analyses easy to conduct and understand would be beneficial and drive	further uniformity among models in future studies.”*
